# Mutations of complement lectin pathway genes *MBL2 *and *MASP2 *associated with placental malaria

**DOI:** 10.1186/1475-2875-11-61

**Published:** 2012-03-02

**Authors:** Ville Holmberg, Päivi Onkamo, Elisa Lahtela, Päivi Lahermo, George Bedu-Addo, Frank P Mockenhaupt, Seppo Meri

**Affiliations:** 1 Department of Bacteriology and Immunology, Infection Biology Programme, Haartman Institute, University of Helsinki, P.O. Box 21, 00014 Helsinki, Finland; 2 Division of Infectious Diseases, Department of Medicine, Helsinki University Central Hospital, P.O. Box 340, 00029 HUS Helsinki, Finland; 3 Department of Biosciences, University of Helsinki, P.O.Box 56, 00014 Helsinki, Finland; 4 FIMM Technology Centre, University of Helsinki, P.O. Box 20, 00014 Helsinki, Finland; 5 Komfo Anokye Teaching Hospital, Kwame Nkrumah University of Science and Technology, Kumasi, Ghana; 6 Institute of Tropical Medicine and International Health, Charité-University Medical Center, Spandauer Damm 130, 14050 Berlin, Germany; 7 HUSLAB, Helsinki University Central Hospital, Helsinki, Finland

**Keywords:** Lectin pathway, Mannose-binding lectin, MBL2, MASP2, Ficolin, Complement, Innate immunity, Malaria, Placenta, Pregnancy

## Abstract

**Background:**

Innate immunity plays a crucial role in the host defense against malaria including *Plasmodium falciparum *malaria in pregnancy, but the roles of the various underlying genes and mechanisms predisposing to the disease are poorly understood.

**Methods:**

98 single-nucletoide polymorphisms were genotyped in a set of 17 functionally related genes of the complement system in 145 primiparous Ghanaian women with placental malaria, defined by placental parasitaemia or malaria pigment, and as a control, in 124 non-affected primiparae.

**Results:**

Placental malaria was significantly associated with SNPs in the lectin pathway genes *MBL2, MASP2, FCN2 *and in *properdin*. In particular, the main African mannose-binding lectin deficiency variant (MBL2*G57E, rs1800451) increased the odds of placental malaria (OR 1.6; permuted p-value 0.014). In contrast, a common *MASP2 *mutation (R439H, rs12085877), which reduces the activity of MBL-MASP2 complexes occurred in 33% of non-affected women and in 22% primiparae with placental malaria (OR 0.55, permuted *p*-value 0.020).

**Conclusions:**

Excessive complement activation is of importance in the pathogenesis of placental malaria by mediating inflammation, coagulation, and endothelial dysfunction. Mutated MBL and MASP2 proteins could have direct intrinsic effects on the susceptibility to placental malaria, in addition to their roles in regulation of downstream complement activation.

## Background

In sub-Saharan Africa, *Plasmodium falciparum *infection during pregnancy is a major cause of maternal anaemia, preterm delivery (PD), low birth weight (LBW) and infant mortality. In areas endemic for *P. falciparum*, 85 million pregnancies occur annually, and malaria-associated LBW in Africa results in an estimated 100,000 indirect infant deaths each year [1-3]. Pregnant, particularly primiparous, women are at increased risk. In pregnant women, parasites expressing specific variants of the *P. falciparum *Erythrocyte Membrane Protein-1 (PfEMP1) adhere to the placental endothelium lining the intervillous space which results in placental sequestration of infected red blood cells. The local accumulation of infected red blood cells and of malaria pigment (haemozoin), i.e. placental malaria, triggers the infiltration of inflammatory cells and a profound pro-inflammatory response [4,5]. This is confronted by an insufficient production of specific antibodies against the parasites and their PfEMP1 binding domain. Only with successive pregnancies, protective acquired immune mechanisms gradually develop and expand, and the disease manifestation declines [6-8].

In conditions of lacking or weak acquired immunity, e.g. early childhood and pregnancy, innate immune responses may play a predominant role in host defense against malaria [9]. Toll-like receptors (TLRs), for instance, are crucial mediators of innate immunity to *P. falciparum *[10], and variant signalling as deduced from functionally relevant single nucleotide polymorphisms (SNPs) has been associated with an increased risk and/or manifestation of malaria in pregnancy [11,12]. Overall, one third of the variability in susceptibility to placental malaria is thought to be due to host genetic factors [13]. These may also include the complement system, composed of three pathways, the classical, the lectin and the alternative pathway.

Mannose-binding lectin (MBL, encoded by *mbl2*) is a serum protein involved in the initiation of innate immune responses by binding to microbial surface oligosaccharides, and activating the lectin pathway. Upon binding, MBL forms a complex with mannan-binding lectin serine peptidase 2 (MASP2) that cleaves C4 and C2 to form the C3 convertase (C4b2a). Subsequent complement activation leads to opsonization and phagocytosis of the target microbes, as well as formation of membrane attack complexes [14]. The role of MASP1 and MASP3 (encoded by *MASP1*) are still debated [15]. An important function of MASP1 is its ability to activate the alternative pathway proenzyme pro-D to active factor D [16]. MASP2 can also form active complexes with ficolins which bind to acetylated carbohydrates or, e.g., to acetylated LDL. Ficolins also recognize deposited C-reactive protein (CRP), and may thus collaborate with CRP in the initiation and control of inflammatory responses [15].

MBL binds to *P. falciparum*-infected red blood cells [17], and may consequently be involved in innate immune responses and parasite clearance. MBL deficiency caused by common SNPs increases the risk of severe malaria although findings are partially inconsistent [18-22]. As for (asymptomatic) placental infection, one recent study failed to show an association with MBL levels or *MBL2 *genotypes [23].

The aim of the present study was to analyse the influence of lectin pathway polymorphisms on the susceptibility to placental malaria and its manifestation. Following a pathway-oriented approach and using Sequenom's MassaARRAY^® ^system, SNPs of *MBL2, MASPs *and *ficolins *(*FCN*) and other important complement system genes (*SERPING1, C3, CFB, CFH, CFP*) were included in a novel "complement-chip". Because the coagulation system may also be involved in adverse pregnancy outcomes [24], we also included respective regulatory genes (*thrombomodulin, thrombospondin, FLT1*).

## Methods

### Patients

A total of 893 women attending for delivery were recruited between January 2000 and January 2001 at the Presbyterian Mission Hospital in Agogo, located in the hyper-to holoendemic Ashanti Region of Ghana. The study protocol was reviewed and approved by the Committee on Human Research Publication and Ethics, School of Medical Sciences, University for Science and Technology, Kumasi, Ghana, and informed consent was obtained from all participants. Diagnostic procedures, malariologic indices and clinical characteristics have been described earlier [11,24]. For the present study, all 304 primiparous women with live singleton deliveries were included. In brief, women were clinically examined, socioeconomic data were documented, and samples of placental intervillous and peripheral venous blood were collected into EDTA. Malaria parasites in intervillous blood were counted microscopically on Giemsa-stained thick blood films per 100 high-power fields (magnification x1,000), and the presence of leukocyte-associated haemozoin was recorded. Placental infection was defined as the presence of *P. falciparum *parasites and/or haemozoin in placental thick blood films. In addition, *P. falciparum*-specific PCR assays were performed [25]. Haemoglobin (Hb) was measured by a HemoCue photometer (Angelholm, Sweden), and anaemia defined as Hb < 110 g/l. Preterm delivery was defined as a gestational age < 37 weeks, on the basis of the Finnström score [26], and low birth-weight as < 2,500 g.

### SNP selection

Based on our previous finding of an association of *MBL2 *polymorphisms with severe malaria in children [22], all lectin pathway genes as well as other relevant downstream complement and alternative pathway genes were chosen for genotyping [27]. Additionally, we included genes related to the coagulation system (*thrombomodulin, thrombospondin*) and pre-eclampsia (*FLT1*). In total, 17 genes were chosen for genotyping (Table 1). For each gene, first, we selected SNPs with assumed relevance based on published data on protein function, activity or disease association. Secondly, we included potentially functional nonsense and missense SNPs, and finally, we also included some intronic, promoter or 3' end SNPs as markers of association. We mainly focused on SNPs with minor allele frequencies > 0.05 in both African and European populations based on HapMap data.

**Table 1:** Genes selected for genotyping.

*HUGO Gene Symbol*	*Gene name *	*SNPs genotyped *	*SNPs analysed for association *
MBL2	mannose-binding lectin 2	5	3
MASP1	mannan-binding lectin serine peptidase 1	8	7
MASP2	mannan-binding lectin serine peptidase 2	11	10
FCN1	ficolin 1	9	7
FCN2	ficolin 2	9	9
FCN3	ficolin 3	3	1
SERPING1	serpin peptidase inhibitor (C1 inhibitor)	5	4
C3	complement component 3	7	5
CFB	complement factor B	5	5
CFH	complement factor H	8	6
CFP	complement factor properdin	6	3
THBD	thrombomodulin	5	1
THBS1	thrombospondin 1	4	3
THBS2	thrombospondin 2	4	4
THBS3	thrombospondin 3	2	2
THBS4	thrombospondin 4	2	2
FLT1	fms-related tyrosine kinase 1	5	4
	**Total number of SNPs**	**98**	**76**

22 SNPs were excluded from genetic association analyses because of low minor allele frequency, poor genotyping quality, or lack of accordance with Hardy-Weinberg equilibrium. Gene symbols and names are given as defined in the HUGO Gene Nomenclature Committee's database

### Genotyping

DNA was extracted from peripheral blood (QIAmp; Qiagen, Germany). Twenty-seven of the samples from 304 primiparous women were excluded from genotyping based on low DNA quality or quantity. Among the 277 samples genotyped, 8 were removed because of a genotyping call rate < 0.9 leaving 269 samples for the final analyses.

SNP genotyping was performed using Sequenom's MassARRAY MALDI-TOF Mass Spectrometry Compact platform and iPLEX Gold chemistry (Sequenom Inc, San Diego, CA) with standard protocols. A total of 98 SNPs were assayed in four multiplexes of 15 to 34 markers each. Eight SNPs that failed to meet a call rate of > 0.8 were removed. 14 SNPs (for which no previous frequency data were available) were excluded due to a minor allele frequency < 0.05, leaving 76 SNPs for the final analyses (Table 1). Genotypes were analysed using Sequenom's MassARRAY Typer version 4.0 software. All data was checked twice manually and all outlying data or low intensity results were removed. The integrity of control and duplicate sample results, as well as negative control samples was checked during the evaluation process.

### Statistical analysis

Analyses were performed with PLINK v1.06 [28], SPSS (SPSS Inc., Chicago, Illinois; release 15.0, 2006) statistical software, and with R language. Hardy-Weinberg equilibrium was tested in infected and non-infected women separately, with the standard Chi-square test, to identify possible genotyping problems. Associations of placental malaria and the SNPs were tested with basic allelic association as well as with several genotypic models (dominant, recessive, and trend included in PLINK). In addition to the main affection status of placental malaria, also other phenotypes were analysed, including low birth weight, preterm birth, maternal anaemia as well as *P. falciparum *positivity by microscopy or PCR from placental or peripheral blood samples. Permutation tests were used to adjust for multiple testing, as well as due to the correlatedness of the tests because of linkage disequilibrium (LD) between markers. Haplotypes were constructed and tested with PLINK using 2-to 6-marker sliding windows over all areas where adjacent SNPs were located less than 10 Mb from each other.

Odds ratios (OR) along with their confidence intervals (CIs) were estimated with PLINK and R scripts [29]. Joint ORs for two loci were evaluated by collapsing genotypes to susceptibility allele carriers and non-carriers, and combining these over both loci, resulting in just four susceptibility classes. This was done in order to avoid sparse class frequencies and thus overly wide confidence intervals.

Finally, interactions were screened with PLINK procedure *epistasis*, which tests all pairs of SNPs separated by at least 1 Mb and in different chromosomes. A *p*-value threshold of 0.001 was used to avoid most false positives. We acknowledge that our data is small for making robust inference on potential interactions, therefore these results should be considered preliminary.

## Results

The characteristics of the 269 primiparous Ghanaian women are shown in Table 2. Fifty-four percent of the women had placental malaria, defined as the presence of parasites or hemozoin in the intervillous placental blood (placental parasitemia, 48%, placental hemozoin, 43%). Placental *P. falciparum*-PCR was positive in 66% and maternal peripheral blood *P. falciparum*-PCR positive in 59% of cases. Maternal hemoglobin and child birth weights were lower in cases with placental malaria than in uninfected mothers (*p *< 0.001 and *p *= 0.023). Gestational age did not differ between the groups.

**Table 2:** Characteristics of 269 primiparous Ghanaian women admitted for delivery

	All	Placental malaria	No placental malaria	*P-value*
Number of subjects	269	145	124	

Mean age, years (± SD)	21.45 (± 3.51)	21.00 (± 3.17)	21.98 (± 3.82)	0.051^1^

Mean maternal hemoglobin, g/l (± SD)	112.6 (± 1.82)	106.3 (± 1.76)	120.0 (± 1.61)	< 0.001^1^

Mean birth weight, grams (± SD)	2760 (± 485)	2698 (± 485)	2833 (± 478)	0.023^2^

Mean gestational age, weeks (± SD)	38.11 (± 2.50)	38.07 (± 2.77)	38.16 (± 2.17)	0.81^1^

*Proportion with *				

> 3 antenatal care visits	0.49	0.44	0.55	0.083^3^

maternal anaemia (Hb < 11 g/dL)	0.41	0.55	0.24	< 0.001^3^

low birth weight (< 2500 g)	0.25	0.30	0.20	0.091^3^

preterm delivery (< 37 weeks)	0.25	0.27	0.23	0.48^3^

*Placental blood samples *				

PCR positive (*P. falciparum*)	0.66	0.95	0.32	< 0.001^3^

Microscopy positive (*P. falciparum*)	0.48	0.88	0.00	< 0.001^3^

Haemozin pigments present	0.43	0.80	0.00	< 0.001^3^

^1^Mann-Whitney U-test

^2^Student's t-test

^3^Chi-square test

A total of 76 SNPs of 17 candidate genes (Table 1) could be subjected to association analyzses. SNPs with at least suggestive association with placental malaria were found on the *MBL2, MASP2, Ficolin 2 (FCN2) *and *Complement factor properdin *(*CFP) *genes (Table 3).

**Table 3:** Single marker results of allelic and genotypic association with placental malaria

			*Allelic associations*						*Genotypic association *	
**Gene**	**SNP**	**Mutation**	**All. freq cases**	**All. freq controls**	***P-value ***	**Permuted *P*-value**	**Odds ratio**	**95% CI**	**Best genotypic model**	**Empirical *p*-value**

MBL2	rs1800451	G57E	0.36	0.26	0.014	0.014	1.60	1.10-2.32	Dominant	0.021

MASP2	rs12085877	H439R	0.11	0.19	0.014	0.020	0.55	0.34-0.89	Allelic	0.015

MASP2	rs1033638	3'UTR	0.36	0.27	0.04	0.022	1.52	1.01-2.26	Trend	0.064

FCN2	rs3128624	Intron 2	0.67	0.62	0.198	0.204	1.27	0.88-1.81	Dominant	0.053

FCN2	rs7037264	Intron 3	0.68	0.64	0.271	0.261	1.23	0.85-1.76	Dominant	0.042

CFP	rs909523	Intron 6	0.57	0.47	0.019	0.029	1.51	1.07-2.13	Trend	0.043

Among these SNPs, MBL2*C (G57E, rs1800451), the main African MBL variant, which is associated with malaria in children [22], was present with an allele frequency of 36% in women with placental malaria, and 26% in controls (*p *= 0.01). The results suggested a dominant effect for an increased susceptibility to placental malaria (Table 3): homozygous or heterozygous MBL2*C was more frequently observed in women with placental malaria (61%, 88 out of 145) than in non-affected women (45%, 56 out of 123) resulting in an OR of 1.8 (95% CI, 1.1-3.0) for the susceptibility allele in our data. MBL*C is significantly associated to microscopy positivity of placental blood samples, whereas other diagnostic tests show non-significant trends in the same direction (Table 4). MBL2*C is not associated to low birth weight, maternal anaemia or preterm birth.

**Table 4:** Association of different phenotypes of *P.falciparum *placental malaria with mutations MBL2*G57E and MASP2*R439H

	MBL2*G57E (MBL2*C; rs1800451)				MASP2*R439H (rs12085877)			
	**Wildtype (GG)**	**Mutation (AA or AG)**	***p-value ***	**OR**	**Wildtype (CC)**	**Mutation (TT or TC)**	***p-value ***	**OR**

Placental malaria	45.5% (56/123)	60.7% (88/145)	0.014	1.84 (1.13-3.01)	57.7% (113/196)	43.8% (32/73)	0.054	0.58 (0.33-0.99)

*Maternal peripheral blood *								

PCR positive	53.7% (66/123)	63.4% (92/145)	0.108	1.50 (0.92-2.45)	62.2% (122/196)	49.3% (36/73)	0.070	0.59 (0.34-1.02)

Microscopy positive	24.8% (29/117)	29.0% (40/138)	0.482		28.9% (55/190)	22.7% (15/66)	0.423	

*Placental blood *								

PCR positive	60.2% (74/123)	70.3% (102/145)	0.094	1.57 (0.94-2.61)	68.4% (134/196)	58.9% (43/173)	0.151	

Microscopy positive	39.0% (48/123)	54.5% (79/145)	0.014	1.86 (1.15-3.05)	49.0% (96/196)	41.1% (30/73)	0.218	

Haemozin pigments present	36.9% (45/122)	48.3% (70/145)	0.064	1.59 (0.98-2.62)	48.0% (94/196)	29.2% (21/72)	0.008	0.47 (0.26-0.83)

Low birth weight	27.0% (33/122)	24.1% (35/145)	0.673		28.6% (56/196)	16.7% (12/72)	0.057	0.51 (0.24-0.99)

Maternal anaemia	35.0% (43/123)	40.7% (59/145)	0.378		38.8% (76/196)	35.6% (26/73)	0.673	

Preterm birth	27.0% (33/122)	23.4% (34/145)	0.571		23.5% (46/196)	29.2% (21/72)	0.344	

**Figure 1:**
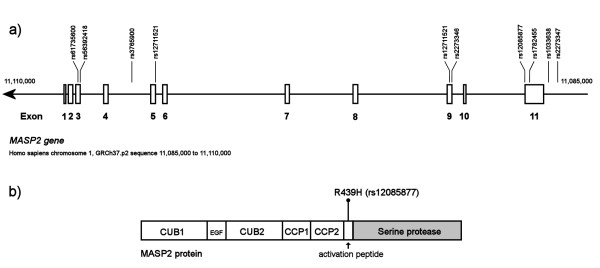
**(a) The genetic map of the region of the MASP2 gene on chromosome 1p36.3-p36.2 is shown (a)**. The SNPs genotyped in this study are marked above the exons. (**b**) The polypeptide chain of MASP2 is composed of an N-terminal CUB domain, followed by en EGF domain, a second CUB domain, two CCP (complement-control protein) domains, an activation peptide, and a serine protease domain

With regard to *MASP2*, two markers, rs12085877 (R439H, *P *= 0.02) and rs1033638 (3' untranslated region, *P *= 0.03), remained significantly associated with placental malaria after permutation tests. These exon 11 variants are located only 930 bp apart (Figure 1a). In this area, one major haplotype including both markers and spanning from rs2273347-rs2273346 conferred increased odds of placental malaria, with an overall *p *= 0.05, and for the haplotype AAGTA specifically, *p *= 0.021 (Table 5). However, the LD between the two major SNPs was surprisingly weak (rs12085877-rs1033638: r^2 ^= 0.037); and most of the risk increase could be attributed to R439H. This amino acid residue is located in the activation peptide of MASP2 (Figure 1b), between the second CCP domain and the serine protease domain, and rhe R439H mutation inhibits the normal function of the MBL-MASP2 complex [30]. When analysed with respect to different diagnostic tests, R439H showed strongest negative association to the presence of malaria pigment in the placenta (*p *= 0.008, OR 0.47, 95% CI 0.26-0.83), but was not associated to microscopy or PCR positivity of the placenta (Table 4). This would suggest a role of the association especially for chronic placental infection. Also, functionality of this mutation was supported by a non-significant trend towards low birth weight in individuals with the 439H variant (*p *= 0.057, OR = 0.51, 95% CI 0.24-0.99).

**Table 5:** Haplotype associations of *MASP2 *and *CFP2 *genes for placental malaria

Gene area	Overall *p*-value^1 ^	Specific haplotype	Prevalence in cases	Prevalence in controls	*P-*value^2^
***MASP2, exon 11 ***					

rs2273347-rs2273346	0.05	AAGTA	0.12	0.19	0.021

***MASP2, 5' end ***					

rs3765900-rs56392418-R99Q	0.05	TGG	0.53	0.21	0.0051

***CFP ***					

rs1048118-rs8177079-rs909523	0.028	ATA	0.08	0.15	0.007

^1^Overall *p-*value for association of the haplotypes in the gene area

^2^*P*-value for association of the specific haplotype

At the 5' end of the *MASP2 *gene, the region rs3765900-rs56392418-R99Q as a whole was associated with placental malaria at *p *= 0.05. Specifically, haplotype TGG was at present in 53% and 21% of women with and without placental malaria, respectively (*p *= 0.0051, Table 5; Figure 1a). None of the markers forming the haplotype were individually associated with placental malaria in permutation tests.

In the *FCN2 *gene, two SNPs showed suggestive associations with placental malaria (Table 3). Located in intron 2 (rs3128624) and intron 3 (rs7037264), they are separated by only 73 bp. These SNPs were not associated to other phenotypes.

Complement factor properdin (*CFP*) is a positive regulator of the alternative complement pathway. It had one SNP associated with placental malaria (rs909523; allele C; *p *= 0.029 after permutation; Table 3). This variant, located in intron 6 of the gene, was not associated with any other assessed malaria phenotype. The region rs1048118-rs8177079-rs909523 was associated at *p *= 0.028, and specifically, the haplotype ATA was protective with *p *= 0.007 (present in 8% and 15% of women with and without placental malaria, respectively).

As MBL forms an active complex with MASP2, the potential interaction between the *MBL2 *and *MASP2 *genotypes was also analysed, to evaluate whether specific allele combinations of the two could render the individual susceptible to malaria. Placental malaria was detected in 39% of the women carrying no risk allels for *MBL2 *(G57E) or *MASP2 *(R439 wildtype), compared to 65% of women with risk alleles at both loci (*p *= 0.007; OR 2.90, 95% CI 1.34-6.48. The increased risk for those with risk alleles at both loci compared to all other individuals gave an OR of 2.13 (95% CI 1.33-3.65, *p *= 0.003). These results suggest an additive effect of both risk alleles, but an excess risk due to interaction could not be shown.

## Discussion

Recent genome-wide association studies on malaria have had difficulties in identifying causal gene variants in African populations due to low LD [31,32]. In the present study, a pathway-oriented candidate gene approach to investigate the role of innate immunity and of the complement system in particular, deficiencies of which are known to increase susceptibility to a wide range of infections [33]. Genetic variants in the *MBL2, MASP2, FCN2 *and *CFP *genes were shown to be potential risk factors for placental malaria in Ghana. These results thus suggest a relevant role for the lectin pathway of the complement system in the human defense against placental malaria.

Functional *MBL2 *mutations interfere with the formation of oligomers and result in low serum levels of high molecular weight MBL and impaired MBL function [34]. Their role in malaria has been addressed in several studies [18-22,35], and a meta-analysis including four studies suggested an increased risk (odds ratio 1.29, 95% CI 1.08-1.53) for malaria in patients carrying the MBL2*C mutation [22]. The strongest association was found in young children [22] supporting the hypothesis that MBL is essential especially in individuals lacking fully developed semi-immunity. In a study from Mozambique, none of ten *MBL2 *SNPs examined were found to be associated with placental malaria [13], but MBL2*C was not included. The absence of association of the other SNPs can probably be explained by the low LD in Africans. These results suggest that the MBL2*C variant is a risk factor for placental malaria, in addition to infant malaria. It remains unclear, however, why such a disadvantageous disposition is maintained at a high frequency in malaria-endemic Africa; respective issues have been discussed elsewhere [21].

Protection from malaria provides an evolutionary benefit in Africa and respective SNPs can be expected to reach polymorphic frequencies. The *MASP2 *mutation *R439H *(rs12085877) is absent in European and Asian populations but according to a previous study [30] and HapMap data, its allele frequency is 9-15% in Sub-Saharan Africa [36]. In the present study, MASP2*R439H occurred in 27% of the women. In contrast to MBL2*C, it was protective against placental malaria, especially chronic infection, and a trend towards protection from low birth weight was discernible. MASP2 with the R439H mutation is able to bind to MBL, but the enzymatic activity of the complex is considerably reduced [30]. At present, conclusive arguments are lacking for the differential impact on placental malaria of two genetic variants which finally lead to reduced complement activation. Thus, it could be possible that these mutations alter the direct intrinsic functions of MBL and MASP2 in addition to the downstream complement activation. MBL has been shown to stimulate phagocytosis and opsonization also in absent of other complement components [37]. MASP2 on the other hand, is capable of promoting fibrinogen turnover directly by cleavage of fibrinogen and indirectly by cleavage of prothrombin, generating active thrombin [38]. MASP2 can, in addition to MBL, interact with ficolins and the balance between formation of ficolin-MASP2 complexes and MBL-MASP2 complex could influence the susceptibility of placental malaria.

Another explanation for MASP2*R439H protecting against placental malaria could involve a curbed production of C5a. Recent work has demonstrated that placental parasitaemia induces increased levels of this potent pro-inflammatory peptide in primiparae [39]. Data from adverse pregnancy outcomes in humans and from murine models of pathological pregnancies suggest that C5a could be an important regulator of placental angiogenesis, and excessive C5a could lead to functional placental insufficiency by impairing adequate vascularization of the placenta [40]. Elevated levels of C5a, however, are considered detrimental for host innate defense including defects in phagocyte and endothelial cell function [41]. Curbed activation in this regard could thus be beneficial.

Alternatively, these findings on a common MASP2 variant show some resemblance with deficiency in the closely related complement receptor 1 (CR1). CR1 is also involved in the activation and regulation of C4, and patients with CR1 deficiency show greatly reduced rosetting, i.e. binding and aggregation of infected and non-infected erythrocytes [42]. Cytoadherence is the key feature of placental malaria [2,8], and small alterations of this process could have substantial clinical consequences. Increased generation of C4b and C3b would thus assist adherence and rosetting of malaria infected parasites in the placenta.

As for the ficolins, two SNPs on the *FCN2 *gene showed a suggestive association with placental malaria. These *FCN2 *variants are located in introns and are thus not likely to be functional themselves. On the contrary, they might be involved in gene expression regulation or splicing, or in LD with the actual predisposing mutations.

Downstream complement amplification of the lectin pathway is normally activated when MASP2 cleaves C4 and C2 to form the C3 convertase (C4b2a). C2 bypass of the lectin pathway is also possible, and involves alternative pathway amplification where complement factor properdin (CFP) and complement factor D (CFD) are involved [43]. This connection of CFP to the lectin pathway could be an explanation of its association to placental malaria. However, the downstream amplification and regulation of the lectin pathway is obviously complex and yet not fully understood.

Excessive complement activation has been suggested to play a role in the pathogenesis of severe malaria, including cerebral malaria as well as severe malarial anaemia, and placental malaria [27]. Through recognition of parasites by MBL and ficolins, the lectin pathway might be able to abort infections and reduce the parasitemic load, but on the other hand be harmful by mediating excessive inflammation, coagulation, and endothelial dysfunction. The novel finding of the protective role of the functional MASP2*R439H mutation makes MASP2 an interesting target for drug and vaccine development. MASP2 inhibitors need to be studied as potential adjunctive therapy for the various manifestations of malaria [44].

## Competing interests

The authors declare that they have no competing interests.

## Authors' contributions

VH designed the study, participated in the statistical analyses and drafted the manuscript. PO carried out the statistical analyses. EL participated in the genotyping. GBA participated in the recruitment of study participants and sample collection in Ghana. FPM coordinated the sample collection and participated in drafting the manuscript. SM participated in the study design and drafting the manuscript. All authors read and approved the final manuscript.

## References

[B1] Desai M ter Kuile FO Nosten F McGready R Asamoa K Brabin B Newman RD Epidemiology and burden of malaria in pregnancy Lancet Infect Dis 2007 7 93 104 10.1016/S1473-3099(07)70021-X 17251080

[B2] Duffy PE Plasmodium in the placenta: parasites, parity, protection, prevention and possibly preeclampsia Parasitology 2007 134 1877 1881 17958923 10.1017/S0031182007000170

[B3] Dellicour S Tatem AJ Guerra CA Snow RW Ter Kuile FO Quantifying the number of pregnancies at risk of malaria in 2007: a demographic study PLoS Med 2010 7 e1000221 10.1371/journal.pmed.1000221 20126256 PMC2811150

[B4] Fried M Muga RO Misore AO Duffy PE Malaria elicits type 1 cytokines in the human placenta: IFN-gamma and TNF-alpha associated with pregnancy outcomes J Immunol 1998 160 2523 2530 9498798

[B5] Moormann AM Sullivan AD Rochford RA Chensue SW Bock PJ Nyirenda T Meshnick SR Malaria and pregnancy: placental cytokine expression and its relationship to intrauterine growth retardation J Infect Dis 1999 180 1987 1993 10.1086/315135 10558956

[B6] Fried M Nosten F Brockman A Brabin BJ Duffy PE Maternal antibodies block malaria Nature 1998 395 851 852 10.1038/27570 9804416

[B7] Duffy PE Fried M Antibodies that inhibit *Plasmodium falciparum *adhesion to chondroitin sulfate A are associated with increased birth weight and the gestational age of newborns Infect Immun 2003 71 6620 6623 10.1128/IAI.71.11.6620-6623.2003 14573685 PMC219546

[B8] Brabin BJ Romagosa C Abdelgalil S Menendez C Verhoeff FH McGready R Fletcher KA Owens S D'Alessandro U Nosten F Fischer PR Ordi J The sick placenta-the role of malaria Placenta 2004 25 359 378 10.1016/j.placenta.2003.10.019 15081631

[B9] Moormann AM How might infant and paediatric immune responses influence malaria vaccine efficacy? Parasite Immunol 2009 31 547 559 10.1111/j.1365-3024.2009.01137.x 19691558 PMC2759986

[B10] Gowda DC TLR-mediated cell signaling by malaria GPIs Trends Parasitol 2007 23 596 604 10.1016/j.pt.2007.09.003 17980663

[B11] Mockenhaupt FP Hamann L von Gaertner C Bedu-Addo G von Kleinsorgen C Schumann RR Bienzle U Common polymorphisms of toll-like receptors 4 and 9 are associated with the clinical manifestation of malaria during pregnancy J Infect Dis 2006 194 184 188 10.1086/505152 16779724

[B12] Hamann L Bedu-Addo G Eggelte TA Schumann RR Mockenhaupt FP The toll-like receptor 1 variant S248N influences placental malaria Infect Genet Evol 2010 10 785 789 10.1016/j.meegid.2010.05.005 20478407

[B13] Sikora M Ferrer-Admetlla A Laayouni H Menendez C Mayor A Bardaji A Sigauque B Mandomando I Alonso PL Bertranpetit J Casals F A variant in the gene FUT9 is associated with susceptibility to placental malaria infection Hum Mol Genet 2009 18 3136 3144 10.1093/hmg/ddp240 19460885

[B14] Wallis R Dodds AW Mitchell DA Sim RB Reid KB Schwaeble WJ Molecular interactions between MASP-2, C4, and C2 and their activation fragments leading to complement activation via the lectin pathway J Biol Chem 2007 282 7844 7851 10.1074/jbc.M606326200 17204478

[B15] Thiel S Complement activating soluble pattern recognition molecules with collagen-like regions, mannan-binding lectin, ficolins and associated proteins Mol Immunol 2007 44 3875 3888 10.1016/j.molimm.2007.06.005 17768106

[B16] Takahashi M Ishida Y Iwaki D Kanno K Suzuki T Endo Y Homma Y Fujita T Essential role of mannose-binding lectin-associated serine protease-1 in activation of the complement factor D J Exp Med 2010 207 29 37 10.1084/jem.20090633 20038603 PMC2812541

[B17] Klabunde J Uhlemann AC Tebo AE Kimmel J Schwarz RT Kremsner PG Kun JF Recognition of *Plasmodium falciparum *proteins by mannan-binding lectin, a component of the human innate immune system Parasitol Res 2002 88 113 117 11936498 10.1007/s00436-001-0518-y

[B18] Bellamy R Ruwende C McAdam KP Thursz M Sumiya M Summerfield J Gilbert SC Corrah T Kwiatkowski D Whittle HC Hill AV Mannose binding protein deficiency is not associated with malaria, hepatitis B carriage nor tuberculosis in Africans QJM 1998 91 13 18 10.1093/qjmed/91.1.13 9519208

[B19] Luty AJ Kun JF Kremsner PG Mannose-binding lectin plasma levels and gene polymorphisms in *Plasmodium falciparum *malaria J Infect Dis 1998 178 1221 1224 10.1086/515690 9806066

[B20] Mombo LE Ntoumi F Bisseye C Ossari S Lu CY Nagel RL Krishnamoorthy R Human genetic polymorphisms and asymptomatic *Plasmodium falciparum *malaria in Gabonese schoolchildren AmJTrop Med Hyg 2003 68 186 190 12641410

[B21] Boldt AB Luty A Grobusch MP Dietz K Dzeing A Kombila M Kremsner PG Kun JF Association of a new mannose-binding lectin variant with severe malaria in Gabonese children Genes Immun 2006 7 393 400 10.1038/sj.gene.6364312 16738667

[B22] Holmberg V Schuster F Dietz E Sagarriga Visconti JC Anemana SD Bienzle U Mockenhaupt FP Mannose-binding lectin variant associated with severe malaria in young African children Microbes Infect 2008 10 342 348 10.1016/j.micinf.2007.12.008 18396436

[B23] Thevenon AD Leke RG Suguitan AL Jr Zhou JA Taylor DW Genetic polymorphisms of mannose-binding lectin do not influence placental malaria but are associated with preterm deliveries Infect Immun 2009 77 1483 1491 10.1128/IAI.01069-08 19139195 PMC2663154

[B24] Mockenhaupt FP Bedu-Addo G von Gaertner C Boye R Fricke K Hannibal I Karakaya F Schaller M Ulmen U Acquah PA Dietz E Eggelte TA Bienzle U Detection and clinical manifestation of placental malaria in southern Ghana Malar J 2006 5 119 10.1186/1475-2875-5-119 17166266 PMC1716171

[B25] Snounou G Viriyakosol S Zhu XP Jarra W Pinheiro L do Rosario VE Thaithong S Brown KN High sensitivity of detection of human malaria parasites by the use of nested polymerase chain reaction Mol Biochem Parasitol 1993 61 315 320 10.1016/0166-6851(93)90077-B 8264734

[B26] Finnstrom O Studies on maturity in newborn infants. IX. Further observations on the use of external characteristics in estimating gestational age Acta Paediatr Scand 1977 66 601 604 10.1111/j.1651-2227.1977.tb07954.x 899778

[B27] Silver KL Higgins SJ McDonald CR Kain KC Complement driven innate immune response to malaria: fuelling severe malarial diseases Cell Microbiol 2010 12 1036 1045 10.1111/j.1462-5822.2010.01492.x 20545944

[B28] Purcell S Neale B Todd-Brown K Thomas L Ferreira MA Bender D Maller J Sklar P de Bakker PI Daly MJ Sham PC PLINK: a tool set for whole-genome association and population-based linkage analyses Am J Hum Genet 2007 81 559 575 10.1086/519795 17701901 PMC1950838

[B29] Medepi.com, R Package for Epidemiological Data and Graphics http://medepi.com/epitools/

[B30] Thiel S Kolev M Degn S Steffensen R Hansen AG Ruseva M Jensenius JC Polymorphisms in mannan-binding lectin (MBL)-associated serine protease 2 affect stability, binding to MBL, and enzymatic activity J Immunol 2009 182 2939 2947 10.4049/jimmunol.0802053 19234189

[B31] Timmann C Evans JA Konig IR Kleensang A Ruschendorf F Lenzen J Sievertsen J Becker C Enuameh Y Kwakye KO Opoku E Browne EN Ziegler A Nurnberg P Horstmann RD Genome-wide linkage analysis of malaria infection intensity and mild disease PLoS Genet 2007 3 e48 10.1371/journal.pgen.0030048 17381244 PMC1829404

[B32] Jallow M Teo YY Small KS Rockett KA Deloukas P Clark TG Kivinen K Bojang KA Conway DJ Pinder M Sirugo G Sisay-Joof F Usen S Auburn S Bumpstead SJ Campino S Coffey A Dunham A Fry AE Green A Gwilliam R Hunt SE Inouye M Jeffreys AE Mendy A Palotie A Potter S Ragoussis J Rogers J Rowlands K Somaskantharajah E Whittaker P Widden C Donnelly P Howie B Marchini J Morris A Sanjoaquin M Achidi EA Agbenyega T Allen A Amodu O Corran P Djimde A Dolo A Doumbo OK Drakeley C Dunstan S Evans J Farrar J Fernando D Hien TT Horstmann RD Ibrahim M Karunaweera N Kokwaro G Koram KA Lemnge M Makani J Marsh K Michon P Modiano D Molyneux ME Mueller I Parker M Peshu N Plowe CV Puijalon O Reeder J Reyburn H Riley EM Sakuntabhai A Singhasivanon P Sirima S Tall A Taylor TE Thera M Troye-Blomberg M Williams TN Wilson M Kwiatkowski DP Genome-wide and fine-resolution association analysis of malaria in West Africa Nat Genet 2009 41 657 665 10.1038/ng.388 19465909 PMC2889040

[B33] Botto M Kirschfink M Macor P Pickering MC Wurzner R Tedesco F Complement in human diseases: lessons from complement deficiencies Mol Immunol 2009 46 2774 2783 10.1016/j.molimm.2009.04.029 19481265

[B34] Larsen F Madsen HO Sim RB Koch C Garred P Disease-associated mutations in human mannose-binding lectin compromise oligomerization and activity of the final protein J Biol Chem 2004 279 21302 21311 10.1074/jbc.M400520200 14764589

[B35] Garred P JS J Quist L Taaning E Madsen HO Association of mannose-binding lectin polymorphisms with sepsis and fatal outcome, in patients with systemic inflammatory response syndrome J Infect Dis 2003 188 1394 1403 10.1086/379044 14593599

[B36] National Center for Biotechnology Information, SNP rs12085877 http://www.ncbi.nlm.nih.gov/snp/?term=rs12085877

[B37] Jack DL Turner MW Anti-microbial activities of mannose-binding lectin Biochem Soc Trans 2003 31 753 757 12887297 10.1042/bst0310753

[B38] Krarup A Wallis R Presanis JS Gal P Sim RB Simultaneous activation of complement and coagulation by MBL-associated serine protease 2 PLoS One 2007 2 e623 10.1371/journal.pone.0000623 17637839 PMC1910608

[B39] Conroy A Serghides L Finney C Owino SO Kumar S Gowda DC Liles WC Moore JM Kain KC C5a enhances dysregulated inflammatory and angiogenic responses to malaria in vitro: potential implications for placental malaria PLoS One 2009 4 e4953 10.1371/journal.pone.0004953 19308263 PMC2655724

[B40] Conroy AL McDonald CR Silver KL Liles WC Kain KC Complement activation: a critical mediator of adverse fetal outcomes in placental malaria? Trends Parasitol 2011 27 294 299 10.1016/j.pt.2011.02.005 21493146

[B41] Ward PA The dark side of C5a in sepsis Nat Rev Immunol 2004 4 133 142 10.1038/nri1269 15040586

[B42] Cockburn IA Mackinnon MJ O'Donnell A Allen SJ Moulds JM Baisor M Bockarie M Reeder JC Rowe JA A human complement receptor 1 polymorphism that reduces *Plasmodium falciparum *rosetting confers protection against severe malaria Proc Natl Acad Sci USA 2004 101 272 277 10.1073/pnas.0305306101 14694201 PMC314175

[B43] Harboe M Garred P Karlstrom E Lindstad JK Stahl GL Mollnes TE The down-stream effects of mannan-induced lectin complement pathway activation depend quantitatively on alternative pathway amplification Mol Immunol 2009 47 373 380 10.1016/j.molimm.2009.09.005 19800125

[B44] Kocsis A Kekesi KA Szasz R Vegh BM Balczer J Dobo J Zavodszky P Gal P Pal G Selective inhibition of the lectin pathway of complement with phage display selected peptides against mannose-binding lectin-associated serine protease (MASP)-1 and -2: significant contribution of MASP-1 to lectin pathway activation J Immunol 2010 185 4169 4178 10.4049/jimmunol.1001819 20817870

